# Health care management in Africa: a debate for future research and agenda

**DOI:** 10.3389/fdgth.2026.1728920

**Published:** 2026-03-02

**Authors:** Albert Attom

**Affiliations:** Loughborough University Business School, Loughborough, United Kingdom

**Keywords:** Africa, digitalization, healthcare, innovation, management

## Abstract

This article provides a critical narrative synthesis of literature on healthcare management in Africa, focusing on two interconnected areas: the impact of managerial capability on shaping integrated healthcare ecosystems and the adoption, implementation, and governance of digital health innovations within diverse health system contexts. Based on health systems strengthening frameworks and socio-technical views on digital transformation, the article explores how managerial skills influence the development and outcomes of digital health initiatives across African settings. Rather than presenting new empirical data, it uses comparative analysis of existing studies to highlight opportunities and ongoing challenges, such as uneven managerial digital skills, resistance to change, system fragmentation, and unintended effects like digital exclusion. The article concludes with a clear and practical agenda for future research and policy, emphasising the vital role of digitally competent managers in fostering supportive organisational cultures, promoting system integration, and ensuring meaningful adoption of digital health innovations by frontline health workers and patient populations.

## Introduction

Effective health management remains a critical and enduring challenge across African healthcare systems, shaped by rapidly growing populations, a high burden of preventable infectious diseases, and rising prevalence of chronic and acute health conditions (1, 2). These pressures are compounded by persistent constraints on financial and human resources (102), alongside declining donor funding and competing global health priorities. Within this context, there is growing recognition that improvements in health system performance depend not only on clinical capacity or technological availability but also on the quality and effectiveness of healthcare management and leadership (3, 4).

Despite the increasing prominence of digital transformation in sectors such as banking, telecommunications, and insurance, its integration into healthcare systems across Africa has been uneven and frequently slower. This has created a gap between the availability of digital tools and their effective use for diagnosing, treating, and managing health conditions (5, 102). Effective healthcare leadership and management are widely recognised as central pillars for achieving universal health coverage, equity, and system efficiency (6). However, weak management capacity continues to undermine health sector reforms, limiting the effective deployment of scarce resources and constraining service delivery improvements at multiple levels of care (7–9).

Central to that strengthening is enhanced managerial capability. Managerial capability plays a pivotal role in knowledge acquisition, effective innovation, policy implementation (10), and enhancing organizational performance and productivity (1, 11, 12). The relationship between managerial skills and technology adoption is particularly notable (1), as these factors are closely linked and contribute to positive organizational outcomes (13).

Recent efforts to advance system integration within African healthcare systems have encountered persistent challenges, frequently linked to shortages of managers equipped with the skills required to lead organisational and technological change (10). Ineffective management has been associated with a range of adverse outcomes, including increased service costs linked to corruption (14), reduced efficiency, low staff morale, diminished patient satisfaction, and poorer population health outcomes (8). Conversely, evidence from multiple settings indicates that strong managerial leadership can improve organisational processes, working environments, and the utilisation of essential services such as maternal and child health care (15, 16).

This article is a Perspective based on a narrative and critical synthesis of existing empirical and conceptual literature. It does not report original empirical data, nor does it follow a systematic review or meta-analysis protocol. Claims advanced in the article are derived from comparative reflection across published studies, policy documents, and theoretical contributions. Where evidence is limited, contested, or context-dependent, this is explicitly acknowledged. It is from this specific perspective that this article examines debates on healthcare management in Africa healthcare systems, focusing on the healthcare ecosystems and the role of digital enabled managers in shaping their development. Limited recognition of healthcare systems as integrated, interdependent ecosystems, and of the managerial capabilities required to sustain them, highlights the need to move beyond technology-centric approaches toward a more management-informed understanding of digital health transformation in Africa.

## Theoretical and conceptual framing

This article is anchored in three complementary theoretical perspectives. First, it draws on health systems strengthening frameworks, which position leadership and governance as foundational building blocks underpinning service delivery, health information systems, financing, and workforce development (17). From this perspective, management is understood as a core system function rather than a peripheral administrative activity. Second, the article engages with socio-technical theories of digital transformation, which conceptualize digitalization as a dynamic process shaped by the interaction of technologies, organizational routines, professional practices, and institutional contexts (18, 19). These perspectives caution against technologically deterministic narratives and foreground the role of managers in interpreting, adapting, and embedding digital tools within existing systems.

Third, insights from healthcare leadership and management competency models inform the analysis of managerial capability, highlighting the importance of strategic, relational, ethical, and digital competencies in navigating complex and evolving healthcare environments (20, 21). Within this framework, healthcare ecosystems are conceptualized analytically as interconnected configurations of actors (patients, healthcare workers, managers, policymakers, and private-sector partners), technologies, governance arrangements, and information flows that collectively shape health service delivery and outcomes. This conceptualization foregrounds interdependence, coordination, and feedback processes across organisational and sectoral boundaries.

## Literature debates

Healthcare management is a fundamental, cross-cutting element within health systems widely recognized as complex social systems; however, the implications of this complexity for management have received insufficient attention in the literature (22). However, evidence on the effectiveness of digital health interventions in African healthcare systems remains uneven and highly context-dependent, with outcomes varying significantly across organisational, regulatory, and infrastructural settings. Healthcare managers' roles are evolving, requiring a broader range of competencies to address emerging challenges and priorities. It is, therefore, critical to ensure that contemporary health managers possess the capabilities necessary to navigate the current healthcare landscape (20). Effective health management is fundamentally centred on the capacity to identify key priorities (23) while working with diverse actors within the health system to deliver (24). As healthcare systems become more complex and digitalized, the competency levels required by healthcare managers to remain effective are an area that remains underreported within the literature.

Digitization has been shown to enhance the cost-effectiveness of healthcare and facilitate the reinvention of healthcare services, making them more dynamic and adaptable to technological advancements (21, 25). The leadership capacity of healthcare managers is a critical determinant of a health system's ability to deliver high-quality services (8). Healthcare managers who demonstrate leadership competencies are essential for driving continuous reforms within healthcare organizations, ensuring the delivery of safe, person-centered, efficient, and effective services at both regional and global levels (24). Studies have demonstrated that healthcare managers' emotional intelligence and organizational communication skills significantly correlate with their effectiveness in healthcare settings (26, 27). Therefore, high-quality management is crucial for advancing healthcare systems, and further exploration of frontline managerial leadership models within the African context is necessary.

## Conceptual Model of Digital Managerial Capability in African Healthcare Ecosystems

**Figure 1:**
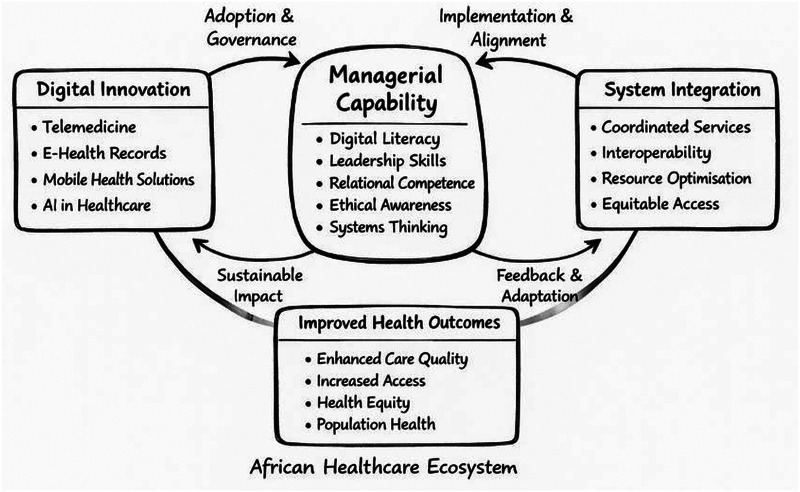
Conceptual model of digital managerial capability in African healthcare ecosystems.

Drawing on health systems strengthening and digital transformation literature, this article proposes a conceptual model that positions managerial capability as a central coordinating force within African healthcare ecosystems (17). Managerial capability is understood as a multidimensional construct encompassing digital literacy, leadership skills, relational competence, ethical awareness, and systems thinking (20, 21). Within digitally mediated healthcare environments, these capabilities shape how innovations are interpreted, governed, and embedded within existing organisational and institutional arrangements. As illustrated in Figure 1, managers play a critical role in mediating the relationship between digital innovation and system integration, influencing whether technologies are aligned with service delivery priorities and broader health system objectives.

Consistent with socio-technical perspectives, digital technologies do not generate value in isolation; rather, outcomes emerge through the interaction of managerial agency, organisational routines, and contextual constraints (18, 19). Where managerial capability is limited, digital initiatives are more likely to remain fragmented, underutilised, or dependent on external actors, constraining their contribution to system integration and sustainability (10, 28). Conversely, managers equipped to lead change and support organisational learning can facilitate coordination, interoperability, and adaptive feedback processes, translating digital innovation into improved health outcomes, including enhanced quality of care, access, equity, and population health gains (29, 30). The model therefore underscores that digital transformation in African healthcare systems is fundamentally a managerial and governance challenge, rather than a purely technological one.

### The role of high competence managers in developing effective healthcare ecosystems

The digitalization of healthcare services and their integration into patient care presents significant challenges regarding the competencies required in both social and healthcare sectors (31). A key factor contributing to the limited success of digital health initiatives across parts of Africa like Ghana is the insufficient education and training of healthcare managers, which directly impacts the quality of care provided (21). Research has shown that many healthcare managers rely on self-directed learning to navigate digital health tools (32), resulting in a fragmented knowledge base and contributing to a lack of consistency across the sector. This gap in digital competencies among healthcare managers is increasingly recognized, particularly in the context of rapidly evolving digital healthcare environments (33). The digital transformation of healthcare requires managers to adapt to the changing demands of the workforce, including staff and managerial roles (34).

Further research (34–36) underscores the need for healthcare managers to receive comprehensive support and training in adopting and implementing digital technologies within their organizations. Effective digital transformation hinges on equipping those responsible for service delivery, such as healthcare managers, with the necessary skills. Enhancing the digital skills of healthcare managers is essential, as they are typically responsible for training and mentoring junior staff in the use of new systems (37). When digital competency is lacking at the managerial due to inadequate training (36), the resulting deficiencies inevitably cascade down through the hierarchical structure of healthcare organizations, especially in Africa, where organizational hierarchies are more pronounced. Thus, healthcare managers must develop the capabilities and contextual competencies required to effectively utilize complex digital tools in a dynamic environment while enhancing digital health literacy to inform strategic and operational decision-making (37).

### The need for digital innovation among primary care organizations

Digital health is increasingly recognised as a driver of healthcare innovation, particularly in supporting patient engagement in primary care (30). Although digital innovation within Africa's healthcare ecosystem is expanding, its reach and scale remain limited, constraining population-level impact (38). Much of this innovation is led by profit-oriented private sector actors, particularly in South Africa, Kenya, and Nigeria, where digital solutions have emerged to address gaps in public healthcare provision (39). These initiatives span access to medical knowledge, quality monitoring, and clinical decision support, and are frequently associated with visionary leadership and effective digital management (40–42).

Extending these innovations beyond the private sector to benefit broader populations remains a significant challenge (30). Achieving scale requires active public-sector engagement and digital managers capable of translating innovation into system-wide practice (43, 102). While strong management is central to developing flexible, patient-centred healthcare systems (10), persistent gaps in digital management capacity continue to constrain innovation and limit the overall impact of digital health initiatives across areas, especially Sub-Saharan Africa (28).

### The need for managerial capacity building towards integrated healthcare ecosystems

Low levels of basic digital competence among healthcare managers raise critical questions not only about skills gaps but also about the capacity of health systems to absorb and sustain digital transformation (21). Although digital health initiatives are often framed as system-enhancing interventions, existing evidence suggests that many managers remain insufficiently prepared to lead within digitally transformed environments, limiting their ability to align technological change with organisational and system-level objectives (28, 44). This misalignment helps explain why digital innovations frequently remain fragmented or confined to pilot projects, despite their technical feasibility. Where managers possess the requisite digital and leadership competencies, transformations are more likely to be embedded and sustained, reinforcing the central role of managerial capability in advancing digital health integration across African contexts (45).

However, the effectiveness of managerial capability is shaped by wider structural conditions. While earlier reviews have identified leadership competencies relevant to digital transformation (46), less attention has been paid to how these competencies are enacted within integrated healthcare ecosystems, particularly at the frontline of service delivery. Digital integration is contingent on consistent funding mechanisms, supportive regulatory frameworks, and alignment with national health policies, without which even competent managers face limited scope to drive system-wide change (46, 47). Collaboration between governments, international organisations, and private-sector stakeholders is therefore necessary but insufficient in isolation; its impact ultimately depends on managers who can translate strategic intent into operational practice, coordinate across organisational boundaries, and sustain integration over time (42).

### Telemedicine and healthcare management in Africa

The Covid-19 pandemic accelerated the adoption of telemedicine platforms across African healthcare systems, positioning remote consultation and prescribing as practical responses to mobility restrictions and service disruption (48). While several studies report rapid uptake and short-term benefits of telemedicine during the COVID-19 period, others highlight difficulties in sustaining these services once emergency conditions receded, particularly where managerial support and integration into routine workflows were limited. Telemedicine enabled healthcare organisations and individual clinicians to deliver care remotely, reducing reliance on in-person visits and partially mitigating infrastructure and workforce constraints, particularly in settings with limited specialist availability and long travel distances (49–53). While countries such as Kenya and Nigeria experienced notable growth in telemedicine services (54), evidence from the pandemic period suggests that telemedicine's rapid uptake was often driven by necessity rather than strategic integration. As a result, although telemedicine became a dominant digital solution for training and consultations during this period (55, 56), its longer-term embedding within healthcare systems remains uneven across contexts.

The literature indicates that variation in telemedicine outcomes is closely linked to managerial capability rather than technological availability alone. Successful implementations highlight the role of managers to align innovation with organisational workflows and professional practice (57). In contrast, managerial resistance remains a significant barrier in many African healthcare settings, where telemedicine is sometimes perceived as undermining the doctor, patient relationship or adding administrative burden without clear benefits (58, 59). These perceptions reflect broader challenges associated with procedural change and role redefinition, which require active managerial leadership to address (60). Where such leadership is absent, telemedicine initiatives are more likely to stall at the implementation stage, reinforcing the view that managerial readiness is a critical determinant of whether telemedicine transitions from a crisis-driven response to a sustainable component of healthcare delivery (57).

### Digital payment and healthcare management in Africa

With the global rise in mobile phone usage, including rapid adoption across East and West Africa, mobile technology has been widely discussed as a potential response to structural challenges in healthcare financing and access (61, 62). Kenya's M-Pesa is frequently cited as a leading example, transforming mobile devices into “mobile banks” and enabling healthcare payments for populations without access to formal banking systems (5, 41, 63). Empirical evidence from Kenya suggests that mobile payment systems have reduced financial barriers for low-income patients accessing healthcare services (9, 64). However, evidence from other African contexts indicates that uptake and impact remain uneven and highly context-dependent, shaped by regulatory environments, digital literacy, and organisational capacity (65). In the absence of such systems, healthcare organisations continue to face increased administrative workloads and higher operating costs associated with cash-based transactions and manual billing (66). While studies from rural settings report improved financial sustainability following the adoption of mobile payment systems, these findings are not consistently replicated across regions, suggesting that positive outcomes cannot be assumed universally (5, 41).

Although electronic payment systems are often presented as broadly beneficial for patients, healthcare organisations, and governments, the strength of empirical evidence varies across settings and implementation models (67). Studies highlight that effective management plays a decisive role in whether digital payment platforms are successfully embedded into routine practice or remain parallel systems with limited operational impact (61, 63). While normative accounts emphasise the role of managers in advocating for regulation, data protection, and inclusive practices, empirical findings suggest that these functions are unevenly realised in resource-constrained healthcare systems (68). Where managerial capacity is limited, digital payment initiatives may reinforce exclusion, generate trust concerns, or face resistance from frontline staff. Expanding digital payment access at scale should therefore be understood as an aspirational objective rather than an empirically established outcome, contingent on the presence of capable frontline managers who can align technology, regulation, and organisational practice (61, 62).

### Electronic health records and healthcare management in Africa

One of the most transformative advancements in global healthcare has been the adoption of electronic health records (EHRs), which hold significant potential for enhancing healthcare delivery efficiency and accuracy (69, 70). The rising emphasis on patient safety and improved outcomes has further driven EHR adoption in many countries (71). Studies have shown that EHR systems improve service quality, efficiency, safety, and patient outcomes (72). EHRs represent an opportunity for healthcare organisations to enhance patient care quality and safety, reduce operational costs, and streamline workplace efficiency (71). Specific benefits of EHR adoption include reduced time and costs related to patient flow (73), remote access to medical records, faster record retrieval (69), and addressing unique regional healthcare challenges (74, 75).

Despite these advantages, African countries have encountered significant challenges in EHR adoption, particularly regarding interoperability. Interoperability is the ability of diverse EHR systems to communicate and exchange data effectively is critical to EHR success (69). Many EHR systems currently in use within African healthcare settings are isolated, creating “islands of information” that limit the seamless flow of patient information between and within different healthcare organizations (24, 57). However, studies have highlighted that health information technology interoperability, while challenging, has seen positive results in hospital settings with effective digital managers who advocate for more connectivity between different departments of the hospital (76). Competent managers and effective digital technology deployment are tightly interconnected and essential for EHR success (77). Skilled managers facilitate EHR adoption by establishing a clear strategic vision, and engaging stakeholders.

### Artificial intelligence and healthcare management in Africa

With a population exceeding one billion, Africa has substantial potential to address its healthcare challenges, with Artificial Intelligence (AI) playing a pivotal role in this transformation (9, 78). AI-driven data science tools already contribute to enhanced patient care and hospital experience (79). The healthcare landscape is evolving rapidly, marked by innovations such as virtual nursing, AI-assisted intensive care, patient education through chatbots, and predictive care tools (80). In Africa, notable AI applications have emerged across various sectors. For example, Nigeria's Hello Tractor (81) improved agricultural mechanization and productivity. AI-powered educational platforms, such as Kenya's M-Shule (103), and Nigeria's Ubenwa have all made use of AI.

Successfully embedding AI in healthcare requires understanding the role of trust in AI as it influences the process of change (82). Implementation strategies across healthcare organizations must be developed to address AI-specific capacity-building challenges, and policies and regulations are essential to guide effective AI deployment. Achieving these aims necessitates collaborative investments among healthcare organizations, government entities, and industry partners (83). Given the novelty of AI and its complex challenges, skilled managers are required to champion the technology and facilitate its acceptance by frontline healthcare workers and patients (104). Without digital managers who understand the potential of AI to change the healthcare landscape in Africa, the effective implementation of AI initiatives will be limited (78).

### Effective digital management

The common thread among these digital innovations is the crucial role of effective implementation strategies attuned to the healthcare ecosystem, creating innovative pathways to enhance current systems (84, 85). Effective digital management must recognize technology's “transformative power” (86) in facilitating digital healthcare innovations (87). By fostering a culture of technological progress, effective managers ensure that digital solutions align with organizational goals and patient needs (88), and promote equity and enhance the efficiency of service delivery (21, 29, 37, 89, 90).

To effectively guide digital health initiatives, managers must possess adequate educational qualifications. Informatics skills and competencies are essential for managers to make informed decisions about digital health services, and these can be developed through targeted education and training (32). For Effective managers to become commonplace within the African healthcare context, there is a need for strategic planning at all levels of the healthcare system towards efforts to allocate resources for training to enhance digital literacy among staff and continuously evaluate performance (14, 46). Strategic interventions to improve the digital competencies of managers will facilitate high-quality care delivery, enhance healthcare outcomes and prepare organizations to adapt to future technological advances, thus positively impacting broader populations (47, 86).

## Future research and recommendations

Strengthening healthcare systems across Sub-Saharan Africa to be more resilient, accessible, and responsive to diverse population needs remains a widely articulated policy objective (53, 91). While existing literature suggests that digitally capable managers can improve decision-making and organisational performance, empirical evidence on the scale and sustainability of such effects across African healthcare systems remains limited (92). This section therefore outlines priority areas for future research and normative recommendations for policy and management development, informed, but not conclusively validated, by existing evidence.

Future research should prioritise context-sensitive studies that examine how regional variation in infrastructure, digital literacy, and service demand shapes the effectiveness of digital health management, particularly across urban–rural divides (83, 93). Comparative and mixed-methods research designs would be especially valuable in exploring how healthcare managers' perceptions of technology differ across cultural and institutional contexts, and how these perceptions influence adoption and implementation practices (94, 95). Such work would move beyond generalised assumptions by generating empirically grounded insights into the interaction between technology, management, and culture.

Further research is also required to examine how leadership, governance arrangements, and supportive policy environments shape digital health adoption, including the role of public–private partnerships in addressing persistent resource constraints. While policy frameworks frequently promote partnership-based models, empirical evidence of their effectiveness remains uneven, underscoring the need for implementation-focused studies that assess how digital solutions are adapted to community-specific values and needs (96–98). Longitudinal research assessing the longer-term impacts of digital health initiatives on health outcomes, cost-effectiveness, and system efficiency would further strengthen the evidence base available to policymakers and practitioners.

### Effective digital healthcare managers leveraging technology in Africa

Normative accounts of digital transformation emphasise the role of managers in fostering data-driven cultures and evidence-informed decision-making through analytics and digital tools (47, 86). However, empirical evidence suggests that such practices remain unevenly distributed, particularly in resource-constrained and rural healthcare settings. Strategic collaboration with technology providers may support the development of accessible and contextually appropriate solutions, yet the effectiveness of such collaborations depends on managers' digital literacy and institutional support (91). Ensuring equitable access to mobile-friendly and low-bandwidth solutions therefore remains a necessary, though not sufficient, condition for improving managerial decision-making across diverse geographical contexts.

### Cross-cultural differences in healthcare managers' views on technology

There is a need to move away from a uniformed approach to management and recognize that cultural differences and norms significantly shape the views of managers on technology (99). These cultural differences can influence aspects such as decision-making styles and openness to adopting new digital solutions, or put simply, ‘Africans see things differently’ (3). By tailoring managerial practices to local cultural contexts, managers can foster more meaningful engagement with technology and enhance its adoption within their communities (100). Management training programs should emphasize cultural diversity, empowering managers to adapt their strategies to local norms, whilst creating “local-global blends” of knowledge to account for global best practices (9). Such culturally sensitive approaches respect the unique aspects of African cultures and improve the relevance and sustainability of digital health initiatives, thereby maximizing their impact on patients.

### A move towards ‘interoperability management’ within the African healthcare system

Fragmented management structures and siloed information systems continue to constrain digital health integration across African healthcare systems. Interoperability management has been proposed as a collaborative approach to enabling secure and efficient data exchange across organisational units (101). While conceptual and case-based studies suggest that networked managers can facilitate organisational learning and information sharing, empirical evidence of system-wide interoperability gains remains limited (69). Interoperability frameworks should therefore be understood as normative models whose effectiveness depends on managerial capacity, institutional incentives, and sustained coordination rather than technical standards alone (47).

### Policy and practice implications

Evidence from the reviewed literature suggests that digital health initiatives in African healthcare systems are most effective when embedded within supportive managerial, regulatory, and organisational environments rather than introduced as standalone technological solutions. However, empirical findings also indicate that policy ambitions frequently outpace implementation capacity, resulting in fragmented systems, pilot dependence, and uneven uptake. Policymakers should therefore exercise caution in assuming that digital technologies will automatically deliver efficiency or equity gains. Instead, digital health strategies should be aligned with investments in managerial capacity, governance coherence, and institutional readiness, recognising that managerial capability is a necessary but not sufficient condition for successful system integration.

From a practice perspective, healthcare managers occupy a critical intermediary role between policy intent and frontline implementation. While normative frameworks often position managers as change leaders, the literature highlights persistent constraints, including limited training, workload pressures, and weak incentives, which can undermine their ability to operationalise digital reforms. Practice-oriented policies should therefore prioritise targeted management development, realistic implementation timelines, and context-sensitive support mechanisms, particularly in resource-constrained and rural settings. For donors, training institutions, and technology partners, this implies shifting emphasis away from technology deployment alone toward sustained investment in managerial learning, interoperability practices, and organisational change processes that can support long-term, system-wide impact.

## Conclusion

This review article synthesised existing literature on healthcare management and digital transformation in Africa, focusing on two interlinked priorities: the development of healthcare ecosystems shaped by managerial capability and the integration of digital innovations within healthcare delivery. The discussion demonstrates that while digital technologies offer potential benefits, their effectiveness is highly contingent on managerial competence, organisational culture, and system-level governance. Rather than presenting digital transformation as an empirically settled pathway to improved outcomes, the article highlights persistent tensions, uneven evidence, and context-dependent effects. It therefore positions managerial capacity building, cultural awareness, and system integration as necessary but not sufficient conditions for sustainable digital health transformation, underscoring the need for future research that moves beyond aspirational discourse toward robust, implementation-focused evidence.

## Data Availability

The original contributions presented in the study are included in the article/Supplementary Material, further inquiries can be directed to the corresponding author.
